# Perioperative Management of Emergency Craniotomes in Children With Cyanotic Congenital Heart Disease: A Case Series

**DOI:** 10.7759/cureus.40840

**Published:** 2023-06-23

**Authors:** Chandan K Dey, Varun Anand, Mussavvir Agha, Habib Md R Karim, Pharanitharan N, Chinmaya K Panda, Manu P Kesavankutty

**Affiliations:** 1 Trauma and Emergency, All India Institute of Medical Sciences, Raipur, Raipur, IND; 2 Anaesthesiology, Critical Care, and Pain Medicine, All India Institute of Medical Sciences, Raipur, Raipur, IND; 3 Anaesthesiology and Critical Care, All India Institute of Medical Sciences, Raipur, Raipur, IND

**Keywords:** emergency neurosurgery, pediatric congenital heart disease, multi-disciplinary care, cerebral abscess, brain swelling, tetrology of fallot

## Abstract

While congenital heart disease is not uncommon, cyanotic congenital heart disease (CCHD) accounts for a minor fraction of them. However, when cyanosis is present, it usually indicates a severe or critical illness. Tetralogy of Fallot (TOF) is one of the common CCHDs, representing 7-10% of all congenital cardiac malformations. Double-outlet right ventricle (DORV) is another CCHD similar to the TOF and associated with decreased pulmonary flow, ventricular septal defect (VSD), and aorta receiving blood from both ventricles. Reduced oxygen arterial saturation and increased viscosity by polycythemia induce focal cerebral ischemia, often in the area supplied by the middle cerebral artery leading to brain abscess. Brain abscesses require craniotomy, which is a major surgery. These patients also often show features of sepsis and increased intracranial pressure. The presence of CCHD further complicates the situation, making perioperative management even more challenging. There are studies in the literature on the management of similar cases, and they report successful management in most of them. However, not all such cases need intensive postoperative management. We present four pediatric cases who had either TOF or DORV and had to undergo craniotomy for brain abscess or ventriculoperitoneal shunt placement. We describe case management and highlight the critical features and cases that require prolonged postoperative critical care management.

## Introduction

Congenital heart diseases are characterized by structural abnormalities that often lead to the mixing of oxygenated and deoxygenated blood. These can be non-cyanotic and cyanotic, and cyanotic ones are also called congenital cyanotic heart disease (CCHD) [1]. Tetralogy of Fallot (TOF) is a common CCHD, accounting for 7-10% of all congenital cardiac malformations [2]. TOF and double-outlet right ventricle (DORV) show similarities in terms of lesions. They are associated with decreased pulmonary flow, ventricular septal defect (VSD), and the aorta receiving blood from both ventricles [3].

Brain abscess is a rare but potentially fatal complication of CCHD. It is often the initial cause of the presentation in developing countries. Reduced oxygen arterial saturation and increased viscosity by secondary polycythemia induce focal cerebral ischemia, often in the area supplied by the middle cerebral artery leading to brain abscess. Right-to-left shunt bypasses of the lungs, which act to filter out the bacteria, are also cited as the cause of infected brain abscesses [4]. Optimal management of brain abscesses involves surgical drainage, culture sensitivity of the pus, and administration of high-dose antibiotics [5]. However, perioperative management, especially anesthesia management, in such patients is challenging due to age, cardiac pathology, and the nature of the surgery. In this report, we discuss a few such cases to highlight the various challenges encountered in the perioperative management of hemodynamics, cyanotic spells, pain, and ventilation and ways to tackle them. Our report is intended to help the perioperative physician in decision-making about on-table extubation and post-anesthesia discharge to a ward or intensive care admissions.

## Case presentation

We present five case scenarios involving four patients who underwent emergency neurosurgical interventions at an academic institute in India. Informed written consent was obtained from the parents/guardians of the children. Institutional research or ethical review and approvals are not required for case reports at our institute. 

Case I

**Figure 1:**
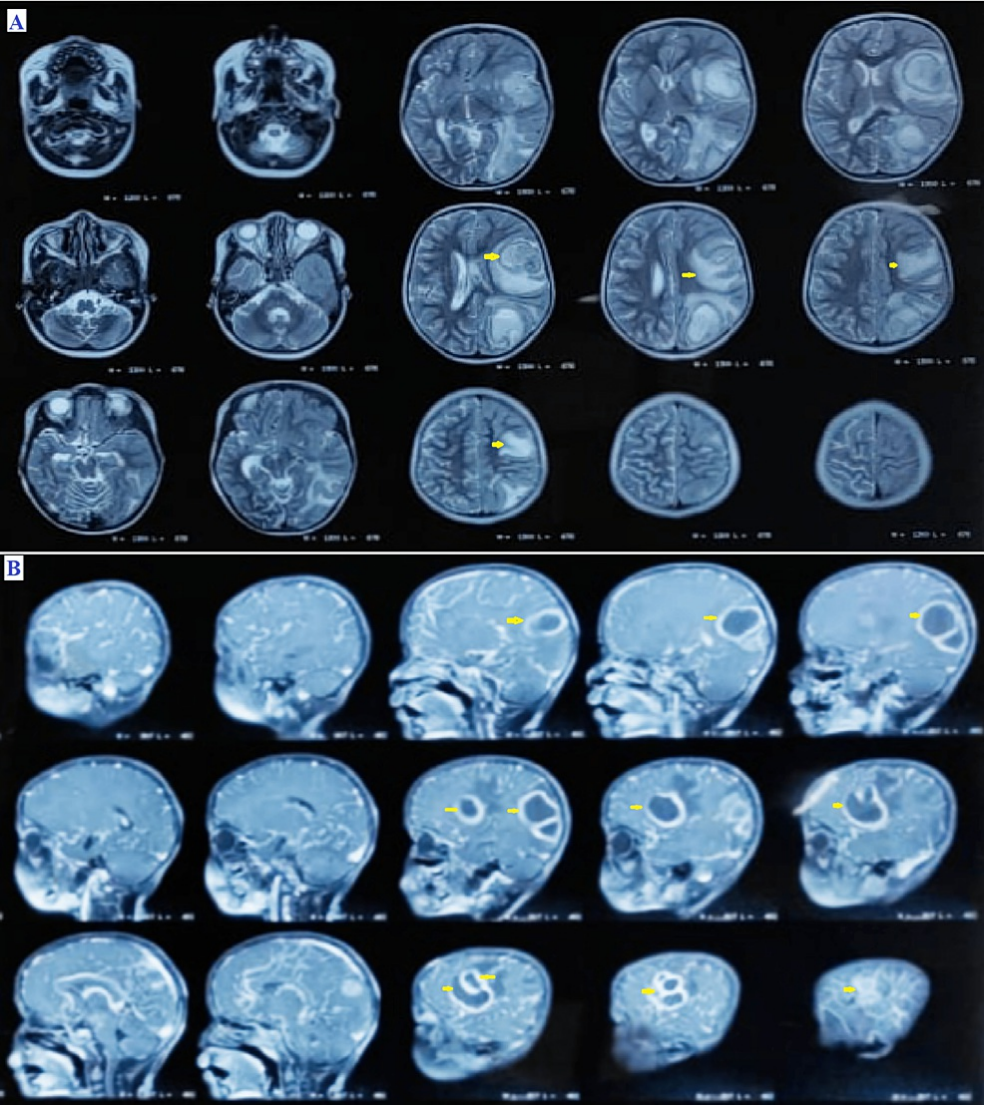
MRI (A: transverse T2 image, B: sagittal T1 image) The images show a large, well-encapsulated, thick-walled peripheral enhancing lesion (arrow) in the left parietal and frontal lobe with brilliantly restricted diffusion, moderate perifocal vasogenic edema, mass effect, and midline shift MRI: magnetic resonance imaging

A three-year-old girl, weighing 8.2 kg (<3 standard deviation for weight for age), presented with fever with chills and rigor for four days, abnormal body movement for three days, and bluish discoloration of lips since seven months of age. She had a history of squatting episodes. She was conscious but irritable with a pulse rate (PR) of 115/minute, blood pressure (BP) of 80/60 mmHg, and respiratory rate (RR) of 36/minute; peripheral oxygen saturation (SpO_2_) was different in all limbs: 97% in right upper, 88% in left upper, 95% in right lower, and 91% in left lower limb, and having central and peripheral cyanosis with clubbing. Apex was within the normal position, and parasternal heave and ejection systolic murmur were present. Neurological examination was unremarkable and showed no hepatomegaly. Brain MRI showed a large, well-encapsulated, thick-walled peripheral enhancing lesion in the left parietal and frontal lobe with brilliantly restricted diffusion, moderate perifocal vasogenic edema, mass effect, and midline shift (Figure 1); 2D-echo showed overriding of the aorta (>50%), small main pulmonary artery (6-7 mm), pulmonary stenosis (PS) with a gradient of 70 mmHg, large non-restrictive VSD with bidirectional shunt, mild tricuspid regurgitation, and no pericardial effusion with normal ventricular functions. Arterial blood gas (ABG) showed a pH of 7.4, PaCO_2_ of 30 mmHg, and PaO_2_ of 45 mmHg on the face mask at 5 L/minute. Her hemoglobin (Hb) was 18.6 mg%, total leucocyte count was 14,490/dl, and platelet count was 1,70,000/dl; renal and liver functions were within normal limits. Chest X-ray showed increased bronchovascular markings, upturned apex, and no pleural effusion.

The patient underwent emergency craniotomy and abscess drainage under general anesthesia (GA). The surgery lasted five hours, and the abscess was drained and sent for culture sensitivity. The patient maintained a saturation of 80-90% intraoperatively. Intraoperative blood losses were replaced with packed red blood cells (RBCs); urine output was approximately 1 ml/kg/hr. Postoperatively, the patient was shifted to the critical care unit (CCU), and meropenem and vancomycin, steroids, and mannitol were administered. She was sedated and ventilated and was weaned off from vasopressors and mechanical ventilation over the next two days. On day four of CCU admission, she was hemodynamically stable, with SpO_2_ of 80-92% on nasal prongs at 1 L/min; oral feeds were started via nasogastric tube, and she was then shifted to the ward.

The patient again presented for ventricular peritoneal shunt placement on the second postoperative week. She was conscious and oriented with PR of 107/minute, BP of 90/60 mmHg, and SpO_2_ of 85-90% on room air. Balanced GA with a multi-modal analgesia technique was employed. Patient hemodynamics were maintained well during the procedure, and she was satisfactorily extubated and put on a facemask O_2_ at 5 L/minute. She was observed in the post-anesthesia care unit (PACU) and weaned off to room air; her SpO_2_ was 88-90% and she was shifted to the ward the same day.

Case II

An eight-year-old boy, weighing 14.7 Kg, with a body mass index (BMI) of 13.1 kg/m^2^, who was a known case of ToF diagnosed one year ago, presented with headache and vomiting for one week and fever for three days. The patient was conscious, oriented, and afebrile on examination, with PR of 64/minute, BP of 107/67 mmHg, and SpO_2_ of 75% on room air; cyanosis and clubbing were present. Neurological, abdominal, and respiratory examinations were unremarkable; apex beat was within a normal position with maximum pan systolic murmur in the lower left sternal border. His Hb was 21 g/dl, platelets were 58,000/dl, urea level was 15 mg/dL, creatinine was 0.51 mg/dL, sodium was 146 mmol/L, and potassium levels were 2.81 mmol/L (intravenous correction was started); his liver function was normal. A 2D-echo showed large subaortic VSD with overriding aorta with bidirectional shunt, severe valvular PS, an infundibular gradient of 81 mmHg, and patent foramen ovale; CT showed multiple brain abscesses predominantly in the right parietal region. Burr hole craniotomy and pus drainage were done. Pus showed 350 WBCs/hpf with 95% neutrophils; no organisms were detected on gram stain and culture. The patient was taken for surgery under the American Society of Anesthesiologists physical status (ASA-PS) IVE; right parietal craniotomy and abscess evacuation were done. The patient was shifted on mechanical ventilation to CCU with FiO_2_ of 0.8. Postoperatively, his SpO_2_ was 60-70%; phenylephrine infusion was continued, and sedation was started; BP and urine output were maintained well. Antibiotics, antiepileptics, and mannitol were started. ABG showed a pH of 7.323, PaCO_2_ of 44 mmHg, PaO_2_ of 32 mmHg, HCO_3_ of 22.5 meq/L, and lactate of 1.97 mmol/L. On day one, the patient was extubated to a high-flow nasal cannula at a FiO_2_ of 90%; he was subsequently weaned off from support and shifted to the ward the next day.

Case III

**Figure 2:**
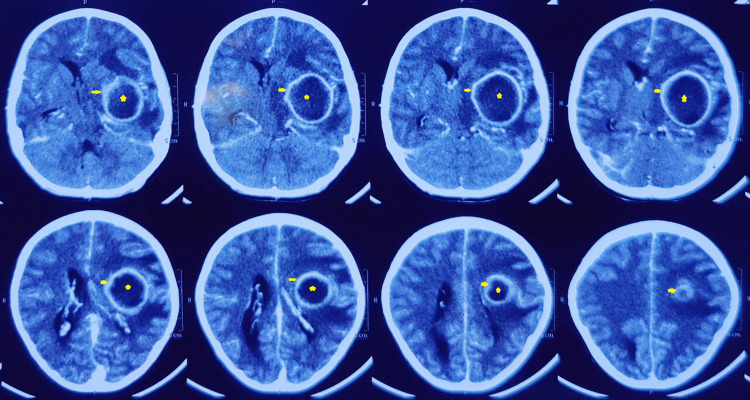
CT (contrast-enhanced) images The images show a large, well-encapsulated, thick-walled peripheral enhancing (arrow) lesion (star) in the left parietal lobe. The mass is effacing the ventricle and causing a minimal mid-line shift CT: computed tomography

A six-year-old girl, weighing 17.4 kg, with a BMI of 12.71 kg/m^2^, a known case of CCHD having VSD with mild PS, presented with a low-grade fever for eight days and headache for which she was investigated and found to have a left temporal brain abscess (Figure 2).

The patient had had shortness of breath and bluish discoloration of lips, tongue, and nails for two years. On admission, she was afebrile with PR of 72/minute, BP of 96/70 mmHg, RR of 21/minute, and SpO_2_ of 85% on room air; pallor and edema were absent, but cyanosis and clubbing were present. She had loud P2 and ejection-systolic murmur; she had GCS of 15, Hb of 21.3 g/dl, TLC of 9,720/dl, platelets of 2,36,000/dl, creatinine of 0.53 mg/dl, sodium of 133 mmol/L, and potassium of 1.52 mmol/L. A 2D-echo showed dilated right chambers, peri-membranous VSD with abnormal left to right shunt, mild pulmonary stenosis, and good systolic function. Intravenous potassium correction was done while the patient was being prepared for drill craniotomy under balanced GA. The procedure lasted for half an hour and was uneventful; the patient maintained 100% SpO_2_ on 50% FiO_2_ and was hemodynamically stable. She was reversed, extubated on the table, shifted to PACU for monitoring, and subsequently shifted to the ward on the same day.

Case IV

**Figure 3:**
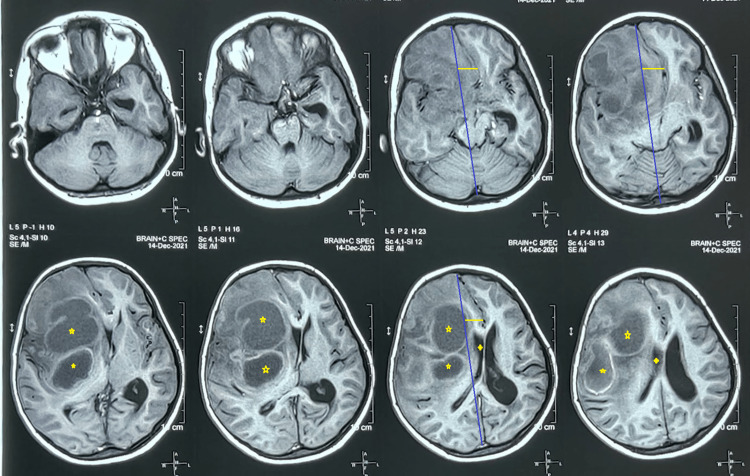
T1 MRI The images show multiple variable-sized well-defined thick-walled lesions (five-point star) in the right frontal lobe and gangliocapsular region; perifocal vasogenic edema and significant mass effect (depicted by straight lines) with partial effacement (four-point star) of the right lateral and third ventricles are also noted MRI: magnetic resonance imaging

A 10-year-old girl, weighing 22 kg, with a BMI of 14.54 kg/m^2^, presented to the hospital with complaints of bluish discoloration of lips while playing since an early age, shortness of breath while walking, facial deviation, and headache for one year. The headache was persistent, progressive, and associated with a few episodes of vomiting. She had a history of one episode of seizure and recurrent respiratory infections. On examination, the patient was conscious and oriented; her PR was 90/minute, SpO_2_ was 72% on room air, and BP was 104/60 mmHg. Pansystolic murmur was present in the mitral area radiating to axillae; laboratory tests showed Hb of 15.1 gm/dl, TLC of 8,780/dl, platelet count of 2,29,000/dl, blood urea of 12 mg/dl, creatinine of 0.41 mg/dl, and electrolytes and liver function tests within normal limits. A 2D-echo revealed DORV, patent foramen ovale with bidirectional shunt, large ostium secundum ASD, dilated right chambers, straddling of the tricuspid valve, large inlet VSD with bidirectional shunt doming pulmonary valve, severe PS (peak gradient 65 mmHg), with good ventricular contractility. Electrocardiogram showed RSR' in II, V3, V4, V5, and V6, and peaked T wave in V2. The patient was started on propranolol 20 mg three times a day. MRI showed multiple variable-sized, well-defined thick-walled lesions in the right frontal lobe and gangliocapsular region. The central part of the lesion showed T2 hyperintense and T1 hypointensity with restriction on DWI, the largest of these lesions being 4.8 x 3.6 x 3.9 cm^3^ in volume. There was gross perifocal vasogenic edema and mass effect (midline shift 10.6 mm) with partial effacement of the right lateral and third ventricles (Figure 3).

Right front-temporoparietal craniotomy and excision of abscesses under general anesthesia were done under the ASA-PS IVE category. The patient had an episode of hypotension during surgery and was started on noradrenaline infusion. Milrinone and vasopressin were subsequently added. By the end of the surgery, adrenaline infusion was also added to maintain hemodynamics. Intraoperative blood loss was replaced with packed RBCs of 350 ml. Around 100 ml of pus was drained. Intraoperative fluid input was 790 ml, and output was 650 ml (urine 250 ml + blood loss). After surgery, the patient was shifted to CCU on mechanical ventilation with sedation and muscle relaxation for further management. On arrival at CCU, her SpO_2_ was 64% on 100% FiO_2_, lactate was 2.0, pO_2_ was 41, and pH was 7.22. On day 0, vasopressin was stopped first in three hours; noradrenaline and adrenaline were also reduced, and she was started on metoprolol for heart rate control, dexmedetomidine, and fentanyl infusion for sedation. The urine output was good. Injection meropenem, vancomycin, mannitol, dexamethasone, and levetiracetam were also administered. Hemodynamics stabilized without support in the next two days, and lactates cleared to 1.3. The patient was extubated and made to wear a facemask; oral feeds were started, and she was shifted to the ward on day four.

All cases were managed by employing balanced GA with multi-modal analgesia. The arterial (femoral) and central lines (right internal jugular) were secured and monitored along with other ASA standard monitoring (continuous five-lead electrocardiography) using a multi-parameter monitoring system, anesthesia gas concentration, capnography measured by the monitor, as well as ABG and input-output monitoring. The management and course of the patients are summarized in Table 1. 

**Table 1:** Summary of patients' perioperative management and critical care course HR: heart rate; HFNC: high-flow nasal cannula; FiO_2_: fraction of inspired oxygen; pO_2_: peripheral oxyhemoglobin saturation; PACU: post-anesthesia care unit; CCU: critical care unit; MACage: age-adjusted minimum alveolar concentration

Timeline/steps	Case I	Case II	Case III	Case I/procedure IV	Case IV
Induction	Ketamine, atracurium	Etomidate, atracurium	Ketamine, atracurium	Ketamine, propofol, atracurium	Thiopentone, vecuronium
Maintenance	Sevoflurane on air + O_2_ (MACage 0.8-1)	Isoflurane on air + O_2_ (MACage 0.8-1)	Isoflurane on air + O_2_ (MACage 0.8-1)	Sevoflurane on air + O_2_ (MACage 0.8-1)	Sevoflurane on air + O_2_ (MACage 0.8-1)
Reversal	None	None	Neostigmine + glycopyrrolate	Neostigmine + glycopyrrolate	None
Analgesia	Fentanyl, paracetamol	Fentanyl, paracetamol	Fentanyl, paracetamol	Fentanyl, paracetamol	Fentanyl, paracetamol
Intraoperative events and management	Tachycardia, hypotension	Tachycardia, hypotension, desaturation	None significant	None significant	Desaturation, hypotension
Intraoperative additional supports	Vasopressin and norepinephrine infusion, esmolol bolus	Phenylephrine infusion, increasing FiO_2_, esmolol bolus	None	None	Norepinephrine, epinephrine, increasing FiO_2_
Postop day-1 supports, management, and events	HR: 100-140 bpm, O_2_ Sat 60-80% on 100% FiO_2_. Vasopressin was titrated off; norepinephrine was reduced, and phenylephrine and esmolol infusion started	HR: 100-125 bpm, O_2_ Sat 79% on 80% FiO_2_, phenylephrine infusion continued	Extubated after the procedure and monitored in PACU on room air. O_2_ Sat 88-90%. Shifted to ward	Extubated after the procedure and monitored in PACU on room air. O_2_ Sat 95%. Shifted to ward	HR: 95-117/minute, Pt on epinephrine, norepinephrine, and vasopressin infusions. Metoprolol for heart rate control
Postop day-2 supports, management, and events	FiO_2_ decreased, norepinephrine titrated off, phenylephrine continued, esmolol stopped, and metoprolol started	Extubated to HFNC at 90% FiO_2_, which could be titrated to 30%, phenylephrine titrated off. The patient kept a negative balance	Stable, no supports	Stable, no supports	HR: 100-115/minute, vasopressin titrated off, epinephrine and norepinephrine on minimal doses, metoprolol continued, dexmedetomidine for sedation. Lactates improved
Postop day-3 supports, management, and events	FiO_2_ further decreased, extubated on facemask at 4 L/minute, phenylephrine stopped	Taken on facemask then nasal prongs at 2 L/minute, SpO_2_ 75-80%			Inotropic support was titrated off; sedation was stopped, extubated to a facemask, and oral feeds started by evening. Metoprolol switched to oral
Outcomes – ventilated days, hospital stay, expired/survived	The patient was stable on day 4, taking oral feeds, and was shifted out	The patient was stable and shifted to the ward on day 4			Vitals maintained well in nasal prongs at 1 L/minute, shifted to the ward on day 4

## Discussion

We discussed four cases that required perioperative anesthesia services five times for emergency cranial surgeries. The cases had different levels of severity in the clinical presentation and other abnormalities like hypokalemia. While anesthesia management of TOF patients for brain abscess surgeries is not uncommon, our heterogenous case management might help the perioperative physicians in extrapolation decision-making on preoperative optimization and postoperative care. TOF is a developmental defect that comprises VSD, overriding aorta, PS, and right ventricular hypertrophy [3]. These patients have right to left shunts, which leads to decreased pulmonary blood flow causing cyanosis at an early age, improper growth, poor feeding, and chronic hypoxia resulting in polycythemia. Patients experience hyper-cyanotic spells during which the right to left shunt increases and pulmonary perfusion decreases as incited by decreased hydration or agitation, which increases dynamic infundibular obstruction [6]. Hypoxemia, hypercarbia, and metabolic acidosis increase pulmonary resistance, decreasing systemic vascular resistance and increasing the shunt. The child is placed in the knee-chest position, ensuring sufficient oxygen, hydration, morphine, and maintaining systolic blood pressure; propranolol is used in severe cases, as it is known to relax infundibular muscle spasms and increase pulmonary flow [7]. Adequate hydration increases venous filling pressure, decreases blood viscosity in polycythemia, and reduces hypercoagulability. DORV is another congenital cyanotic heart anomaly in which both aorta and pulmonary artery arise entirely or predominantly from the right ventricle [8]. It occurs at a rate of 0.03-0.14 per 1000 live births and constitutes about 1% of all CHD [9]. It is almost always associated with VSD, but PS, ASD, PDA, atrioventricular canal, subaortic stenosis, coarctation, and hypoplastic aortic arch are also frequent [10]. The child presents with cyanosis, congestive heart failure, failure to gain weight, frequent respiratory infections, and polycythemia.

The anesthesia plan before surgery was to keep the patients calm and avoid any stressors; we took help from the parents as the children were overly attached to their mothers. The objective was to avoid any cyanotic spell in the perioperative period. During surgery, we tried to avoid hypoxemia [11], acidosis [12], and hypercarbia [13], maintaining systemic vascular resistance (SVR) as far as possible, minimizing pulmonary vascular resistance, maintaining euvolemia, and meticulously avoiding air bubbles in IV lines to prevent paradoxical air embolism [7]. Cases were mostly induced with intravenous ketamine, as it can maintain SVR and is hence the favored drug for these cases over inhalational induction [14]. Etomidate is another agent associated with good cardiovascular stability. Phenylephrine was used predominantly to assist in hemodynamics, as it is an alpha-agonist that can increase SVR and is effective against hyper-cyanotic spells. Vasopressin and norepinephrine were also started intraoperatively to maintain SVR when an additional agent was required. Esmolol has many benefits in patients with hyper-cyanotic spells; it decreases heart rate, increases filling time, relaxes infundibular spasm, is short-acting, and thus can be titrated easily, and has been used effectively before [15]. In the postoperative period, the plan was early extubation to decrease the use of sedation and prevent noxious stimuli to the patient to prevent hyper-cyanotic spells. Norepinephrine and vasopressin were titrated off; phenylephrine infusion was started, and intravenous hydration was maintained with a continuation of esmolol infusion. Phenylephrine can increase SVR without increasing heart rate [16]. After extubation, esmolol infusion was titrated off, and the patient was started on metoprolol orally. Antiepileptic mannitol was continued in the perioperative period. Managing cyanotic heart disease patients for non-cardiac surgery and also in the postoperative period has always been challenging. This report aims to share our experience and positive results with this management.

Inhalational agents cause vasodilatation, thus reducing SVR and consequently reducing left to right shunt. Hydration and inotropes that increase SVR can be started. Sympathetic stimulation like intubation response can be reduced by fentanyl and by providing adequate analgesia and ensuring depth of anesthesia during surgery. One of our patients went into shock that was only responding to inotropes. In the postoperative period, as urine output increased, shock due to congestive heart failure resolved, inotropes were titrated off, blood lactates were reduced, and the patient could be extubated. Oxygen requirement also decreased as pulmonary circulation increased. Thus, optimizing intraoperative fluids is essential.

Although we observed increased severity of the TOF as found clinically by lower saturations, cyanotic spells and electrolyte abnormality were present in the cases which required perioperative intensive care management, and the observation was limited to just four cases. Furthermore, an association could not be ascertained from such reports. We will require prospective studies for the same.

## Conclusions

Emergency craniotomies and VP shunt placement in patients with CCHDs are challenging but can be well-managed at tertiary care hospitals with CCU backups. Although our case series did not touch on independent risk factors or parameters predating postoperative CCU admission or prolonged hospital course, patients' preoperative condition, the severity of the CCHD, and lower preoperative PaO_2_ or SpO_2_ appear to be associated with a long and turbulent postoperative course. Balanced GA using modern inhalational agents, newer sedatives like dexmedetomidine, short-acting opioids like fentanyl, and a multi-modal analgesia plan, appears to be safe and effective in managing such patients.
